# A temperament for learning: The limbic system and myelomeningocele

**DOI:** 10.1186/1743-8454-1-6

**Published:** 2004-12-10

**Authors:** Behroze Vachha, Richard C Adams

**Affiliations:** 1 Pediatric Developmental Disabilities, Texas Scottish Rite Hospital for Children, Dallas, TX 75219, USA; 2 Department of Pediatrics, University of Texas Southwestern Medical Center, Dallas, TX 75219, USA

## Abstract

This article was the winner of the triennial Casey Holter Memorial Prize awarded by the Society for Research into Hydrocephalus and Spina Bifida, 2004.

**Abstract:**

This essay explores the link between the limbic/hypothalamic systems within the complex conditions of hydrocephalus and myelomeningocele. Acknowledging the neuroanatomical and neuroendocrine risks inherent in the developing brains of these individuals, we focus on the converging components of temperament, cognition, and language.

## Introduction

Children with myelomeningocele (MM) do not easily fit into stereotypical profiles. Professionals, families, and inter-disciplinary teams, devoted to the support of these individuals and the reduction of secondary disabilities, see daily testaments to individual variations. Clinical commonalities among those with MM are frequently noted and perhaps too often – and too simplistically – considered *inherent *to the condition. Behavioral characteristics have long been one such category for easy generalities. As modern investigators study the underlying keys to cognitive and behavioral disabilities, theories related to cortical development and differentiation have offered insightful possibilities [1]. Much remains to be discovered in the realm of behavior, emotions, and personality profiles within this group and the biologic differences that may explain them.

This essay is submitted with the suggestion that in our ongoing quest to understand the learning and behavioral characteristics in youth with MM, focus might reasonably be placed on cellular and neurobiological mechanisms specifically related to the limbic and hypothalamic systems. Given the developmental shifts in neuro-architecture in the fetus and infant with MM, we offer the limbic system with its cortical, and brain stem interconnections, and particularly its close association with the hypothalamic region, as an area wherein many of the phenotypic commonalities may arise.

Similarities and variations in temperament profiles among children with MM present an opportunity to explore and enhance our understanding of this ontologically old but, as yet, not fully understood portion of the brain. Temperament patterns among this group, differing from other cohorts, may be reflective of altered integrity within this intricate neural system. The converging components of temperament, memory, and language in this group of children with developmental differences may help the researcher and the clinician to better address the so-called *behavioral *issues that impact academic learning.

We will re-visit established and emerging information related to the limbic and hypothalamic systems. We will review reports from recent studies on language and on temperament among children with MM, and comparative work focused with different at-risk populations of children. These will be considered along with other clinically relevant phenotypic findings among children with MM that have clear links to the limbic and hypothalamic systems.

## Le Grand Lobe Limbique

The limbic system consists of structures reminiscent of the old mammalian brain corresponding to that of the so-called higher mammals [2,3]. The various components of the limbic system influence a diversity of functions that are integral to the cognitive aspects of autonomic, affective, and sexual behavior [4]. Components of the system and their general activities that might be expected to impact memory and/or temperament include the following:

***Amygdala ***– little almond shaped structure near the temporal pole. It receives catecholamine and 5-HT containing projections from the brain stem. Its most prominent projection (stria terminalis) runs in the wall of the lateral ventricle, ultimately connecting to the hypothalamic center. Along with other behaviors (particularly sexual), it mediates the major affective activities describe as *fear *[3,5,6].

***Anterior thalamic nucleus ***– associated with variations in emotional reactivity [3].

***Cingulate gyrus ***– located on the medial side of brain near the corpus callosum. Implicated in encoding and retrieval of semantic and episodic memories, attention, drive and pain perception [7].

***Hippocampus ***– formed from the inferior portion of the temporal lobe into the lateral ventricle. It is involved in memory, especially long-term memory. When both hippocampi are completely ablated, nothing can be retained in the memory. The intact hippocampus allows comparison of the conditions of a present challenge with similar previous experiences. This process is central to the ability to make choices [3,8]. This can be critical for survival in the wild – or perhaps survival in the classroom.

***Entorhinal, subicular cortices ***– are implicated in spatial memory storage, connect hippocampus to remainder of temporal cortex [2].

***Septal nuclei ***– a subcortical target of hippocampus [2].

***Mamillary complex ***– is the major limbic-hypothalamic area [2].

From a cellular and structural aspect, the proximity of limbic structures to the ventricles hints at the possibility of neuropathological changes in these structures either directly through mechanical compression effects of the dilated ventricles, or indirectly via alterations of metabolic pathways in children with MM [9-11]. Structural changes, and potential functional sequellae, may also arise through deficiencies in the developmental process when HC occurs in the fetus. Behaviorally, an emerging body of neuropsychological data suggests a critical relationship between these structures, their functions, and their relationship to emotion, memory, and learning.

## Tell-tale signs of limbic influence: Learning and Temperament

Much has been written over the past decade describing phenotypic profiles related to executive functions among youth with MM. While the term executive function continues to be somewhat enigmatic, it has generally been used to encompass a set of abilities including: sustained focused attention and organizational skills, goal-directed behaviors, flexibility with novel situations, generation of unique plans, and working memory functions [12,13]. Long relied-upon anecdotal concepts (short attention spans, poor organization skills, and memory problems) have given way to more controlled studies [14-19].

Language skills evaluated in recent research described both language production and comprehension within theoretically grounded language subsystems [20-30]. From this, a modal profile for MM has been proposed: strengths in syntax and lexicon; and weaknesses in pragmatic communication, making inferences, and understanding nonliteral language [31]. Research supporting this modal profile for children with MM (especially as it relates to higher-order language skills) is based on their inclusion within the larger heterogeneous samples of children with hydrocephalus (HC) whose conditions were associated with multiple etiologies, both congenital and acquired (e.g. refs. [22,30]). Recent studies of a large sample of children with shunted HC and MM *only*, demonstrated that these children scored lower than age-matched controls in all areas of language skills – even in the more basic lexical semantic skills which, have long been considered "strengths" [26,29]. But two areas provided the children with particular difficulty: supralinguistic skills (making inferences and understanding nonliteral language) and pragmatic judgment.

Cognitive skills of attention/executive function and memory are deeply embedded within acquisition and appropriate application of language [32,33]. The ability to make inferences and understand ambiguities (both supralinguistic tasks) involves the integration of previously learned world and linguistic knowledge with the new knowledge presented [34,35]. For a pragmatically competent communicator, successful language outcomes depend on adequately functioning systems of memory (encoding and retrieval of experiential and linguistic knowledge), attention (ability to focus on the presented communicative task), and problem solving (including the ability to judge and utilize social situations). The few studies that measured memory function in mixed samples of children with MM and HC have demonstrated memory deficits in encoding and retrieval both for verbal and nonverbal information [16,18]. Similarly, current research seems to suggest a prevalence of attention/executive deficits in children with MM [14,15,17,19,36,37].

A more than moderate interaction between the successful development and function of the limbic structures and successful learning experiences among children with MM seems likely. Structural or functional malformations of this system should impact the unique learning and cognitive profiles of these children.

Prefrontal regions have been identified as critical in functions of memory, language and logical reasoning [38-40]. Animal models and clinical evidence suggest the integral role of the limbic system. The uni-modal and poly-modal inputs from neocortical association areas (including prefrontal), important for information processing, storage, encoding and retrieval, all rely on competent structures within the limbic system [8,41-46].

How might these facts relate to our children with MM? Shortly after the neural tube is formed, its caudal portion develops into the spinal cord and the rostral portion into the brain [3]. Of the three early brain vesicles, the forebrain gives rise to the telencephalon and diencephalon from which structures of the limbic system and the hypothalamus arise. The limbic system, arising from this phylogenetically ancient region of the brain, incorporates the hippocampus, the amygdala, and numerous receptor sites for adrenal and endocrine neuromodulators.

MM, resulting from a developmental disruption of this normal neuroembryogenic process, is commonly characterized by its more visual and tangible manifestations: defects within the bony vertebra, associated impairment of the spinal cord, and the functional motor and urological deficits. The clinical manifestations of underlying neurodevelopmental aberrations centrally (the Arnold-Chiari II malformation, ventricular variations, callosal variations, and associated migrational anomalies) are frequently as disabling as the more obvious motor deficits. Structural and functional differences can result from developmental and/or mechanical disruptions in the formative stages – prenatally or thereafter. These central nervous system anomalies, often accompanying the MM, would be expected to influence behavior and learning in these children [e.g. [47,48]].

Developmental disruptions such as those related to MM can also provide "spin-off" developmental deficits related to anatomy and physiology arising from structures that form the limbic system and hypothalamus. Similarly, the potential exists for the developing HC, with its cerebrospinal fluid blockage within and around the brain, to impact the architecture of the emerging limbic system and its neuronal projections.

For example, vacuolization and degeneration of neurons in the hippocampal formation have been observed both within hydrocephalic rabbits and humans [9]. Dendritic changes were observed in the hippocampus of neonatal rats with kaolin-induced HC [10], and HC exacerbated hypoglycemic injury in rat hippocampal cells [49]. Indeed in a rat model of kaolin-induced HC, the resultant impairment of glucose metabolism was first observed in the CA3 region of the hippocampus [50], suggesting this area is metabolically vulnerable to dysfunction. Further support of limbic involvement in HC comes from a recent study that reports damage to the fimbria/fornix of the hippocampus from autopsy-acquired brains of humans with chronic HC [51].

Clinical evidence from patients, and recent brain imaging studies suggest lesions of the right hippocampus result in spatial memory deficits while left lesions impact verbal memory [3,8,52]. Thus, resultant left and right CA3 dysfunction potentially contributes to the verbal and nonverbal memory deficits noted in children with MM and HC. Further, findings by Dolan and Fletcher [53] provide evidence that the left hippocampus is active in encoding of episodic memories by registering and processing novel verbal material, the end-product being the so-called memory trace or engram. The inability of children with MM and HC to respond efficiently to novel stimuli is well documented [54,55].

Anatomical and central molecular dynamics involved with these language and memory deficits remain critically important areas for future research. Equally intriguing are those – or other – molecular substances and pathways that mediate the *response *to the perceived frustrations associated with functional deficits, which come into play daily and hourly. What underlies and mediates the *style *of behavioral responses? In other words, how does the child's temperament phenotype become an integral part of the clinical management, and neurophysiological understanding of learning differences among children with MM?

This task of describing individual differences among individuals within a society is ancient in its origin. Early Chinese tradition offers a view of human nature differences based on a dark, female, earthly force (*yin*) and the accompanying active, light, heavenly force (*yang*). The great 2^nd ^century C.E. Roman physician, Galen, conceived a system of balanced equilibrium of four basic humors, which allowed the physician clues as to the nature of an illness by his awareness of personality traits. This system, based on yellow or black bile and phlegmatic or sanguine natures, represented a culmination of prior ideas (Hindu, Greek, Chinese, others) and provided the understanding of the relationship of temperament to health and function for more than a millennium [56].

More modern scientists have provided differing slants regarding temperament characteristics of children. The list includes Bates, Buss, Carey, Chess and Thomas, Goldsmith, Kagan, and Plomin [56-61]. The nature of temperamental categories, taking the various researchers' ideas into account, seems to embody four qualities described by Kagan: 1) variability among individuals, 2) relative stability over time and situation within the individual, 3) under some genetic influence, and 4) emergence early in life [56].

Temperament has been described in typically developing children as the "how " of behavior distinct from *ability *or "what" of behaving, and *motivation *or "why" of behavior [60]. One recurring theme among the conceptual descriptions of temperament remains the notion of multiple behaviors, along a continuum, which combine to interact either favorably or unfavorably with the surrounding environment. The nature of these behaviors is not at the level of psychopathology. Rather, the potential for pathologic development lies within the *process of the interaction *– the "goodness of fit" – between the environment and the individual's behavioral responses. Consonance between the child and his/her environment potentiates optimal development and supportable teachable moments [62]. Conversely dissonance between the child's capacities, and style of behavior and the environment demands result in maladaptive functioning. Such was the case with Annie.

## Annie's Story

Annie is an attractive little girl, eight years old, and with abundant red-haired curls. Her diagnosis of MM and shunted HC requires part-time wheelchair use as she attends a public school and participates in recreation activities in the community. Intelligent and engaging, she has, nonetheless, met frustrations during her days at school. Variably described as shy, capable, anxious, and/or self-doubting, teachers and family members know of Annie's apparent emotional lability.

Recent academic testing reconfirmed her cognitive strengths with a full scale IQ in the above-average range. Language evaluation showed "relative strengths" in lexicon and syntax, lesser scores in pragmatics, and significant problems with higher-order language, and cognitive tasks that required quick retrieval of stored information. Of note, during the initial testing, Annie became tearful quite quickly. Her negative responses related to the new tasks, new environments, and her persisting fear of "not being able to finish" (on any given activity). The examiner, familiar with children with MM, recognized the triggers and the responses before her. By careful explanations, negotiating cues to assure Annie of her successes in the testing, and agreements for "breaks" as needed, Annie calmed, warmed to the tasks at hand, and completed the testing successfully in the allotted time.

The developmental pediatric team was recently notified by the family that Annie was experiencing episodes at school of skin flushing, sweating, sensations of nausea, and emotional stress. On one occasion, urinary incontinence accompanied the episode. These symptoms were eventually noted to be related to those (infrequent) occasions when the teacher felt it necessary to enter a note onto Annie's personal folder for parent review. Beginning hints of school avoidance prompted the parents' concern.

Academic challenges and barriers as in the story of Annie are quite common among youth with MM. Layers of seemingly separate but simultaneous factors – cognitive, memory, language, temperament, physical – can elevate even daily and mundane tasks into sources of stress not experienced by the typical classmate. Recent studies of youth with MM and shunted HC, ages 5–12, demonstrated clusters of findings different from those classically described by Chess and Thomas ("easy", "difficult", or "slow to warm") or by Kagan ("inhibited" or "uninhibited"). Children with MM in these studies were reported to be significantly different from normative profiles in five areas: 1) adaptability – less; 2) distractibility – more; 3) approach – guarded; 4) persistence on task – less; and 5) predictability – less [55]. Given these temperament characteristics in a child with MM, perhaps related to the unique differences in both central molecular dynamics and neurodevelopmental anatomy, responses such as Annie's begin to be understood.

While the focused study of the biological basis for temperament dates to mid-20^th ^century, only recently have physiologic variables and temperament categorical descriptors been paired for critical comparisons. These investigations stem from relatively firm notions about the anatomy and physiology of the central nervous system. Researchers have tracked differing patterns of social response, and encoding of emotional memories to the limbic system, and particularly amygdala [63,64]. The amygdala has been implicated in social cognition, involving adequate recognition and judgment of facial expressions [3,65]. Preliminary studies within our institution reveal our patients with MM and shunted HC had difficulty understanding nonverbal facial cues and expressions of annoyance (explaining in part their poor pragmatic skills). Opioid-mediated inhibitory activities, or responses to the dozens of neurotransmitters or neuropeptides both have genetic implications and biochemical importance as they potentially affect excitability centrally within the amygdala or connecting structures [56]. Potential changes in functional outcomes might be anticipated.

These limbic structures – and their vulnerability in the developing brain affected by MM – offer fertile ground for clinical exploration in the broader study of cognition, memory, and temperament. The segregation of emotion, temperament, language, and cognitive performance is difficult to accomplish in the typical child; it is decidedly more complex in the child with MM and HC who has co-existent ventriculomegaly from early gestation, mid-brain variations, and cerebellar differences.

What then are the capacities and activities of these central nervous system components – the limbic and hypothalamic systems – that are typically relied upon for daily function, and which might have significant impact on those outward actions and responses which we categorize as temperament? Emotional triggers, goal direction for problem solving, assimilation of information from the various senses (vision, touch, auditory) – all of these relate to the anatomy and associated function of the limbic system (notably the amygdala), and the closely related hypothalamic region. Evidence from human and animal studies indicates that the amygdala intervenes between the regions concerned with the somatic expression of emotion (hypothalamus, brainstem) and the neocortical areas concerned with conscious feeling [3].

Similarly, the hypothalamus is a central player in the integration of information. For the specific areas throughout the neocortex to finalize and actualize those processes described as "executive functioning", regulation of stimuli from the environment and peripheral systems of the body is aided through hypothalamic function. Some actions might well be expected to impact behavior we characterize as components of temperament.

The anatomy of the hypothalamus is important when considered along with the variations commonly noted in our individuals with ventriculomegaly and Chiari malformations. It lies on the ventral surface of the cortex. The third ventricle divides the hypothalamus and lies in close proximity to hypothalamic projections [66]. Disturbances or disruptions in these structures due to HC would not be unexpected. Generally, signs or symptoms associated with hypothalamic dysfunction might include: disorders of satiety, appetite and/or caloric homeostasis; sexual dysfunction including precocious puberty or hypogonadism; central autonomic disorders (sleep and consciousness regulation, gastrointestinal function, thermoregulatory control, sphincter disturbance); and affective variations [3,66]. Corticotrophin releasing factor, related to the paraventricular nucleus, is integrally involved in the management of autonomic centers within the brainstem and, via regulation of corticosteroids, with stress adaptation [3].

The effects of HC on neuronal pathology and function in these regions have been demonstrated in both human studies and animal models [10,49,50,67]. Such variations in anatomy and fetal development of the midbrain structures – areas of tremendous variations among individuals with MM – could play important roles in the ultimate behavioral phenotypes described by temperament. For example, in the temperament profile of the MM cohort described above, attention was a significant outlier. Mirsky [68] has reminded us of the multi-component nature of attention. The nature of the neuropsychological model of attention depends upon the data used to generate it. The network involved in the distribution of attention to extra-personal targets implicates cortico-limbic-reticular and hypothalamic circuits; damage to either impacts the attention process [68]. The ultimate functions completed within cortical regions are clearly important. But dysfunction in the limbic and hypothalamic regions seems intuitive within this population. They can be measured in some instances (particularly neuroendocrine), and they are consistent with the underlying biologic bases of temperament variation as posited by Kagan and others.

## Temperament as a Component for Assessment of Learning in Youth with MM

Annie's tale is not unusual for our children with MM and shunted HC. Clinicians and researchers alike have experienced stories of lesser or more severe impairments. For the parents and teachers working daily with children such as Annie, an understanding of the normal process of neuro-development leads to clearer understanding and a better differentiation from the abnormal. The research and subsequent writings of T. Berry Brazelton have provided ready information to parents of typically developing children by blending the Gesellian milestones of development with the temperament concepts as outlined by Carey, Chess, and Thomas [69]. The interplay of cognition, temperament, language, executive function, given the underlying neuro-endocrine/central molecular dynamics that accompany MM, offers a greater challenge to clinicians, teachers, and parents.

For the classroom, identification of stressors and methods to minimize autonomic over-response can be as critical to successful learning as the understanding of memory strengths, or language problems. As in our story, Annie demonstrated persistence to task, willingness to approach novel situations, positive mood, and the ability to adapt to the testing situation – all temperament domains with which she has struggled. The "goodness of fit" between Annie and the examiner was critical before *any *progress could be made in better understanding her relative academic and learning strengths. The autonomic dysfunction, manifested in her excessive sweating, flushing, and urinary incontinence in school, only served to heighten both the emotional and physical symptoms.

The differences in temperament profiles among children with MM are unlike those in other diagnostic cohorts of children with special health care needs [70,71]. Hughes et al. [72] have described temperament patterns in preterm infants over the first year of life. Descriptors of temperament characteristics differed from that seen in typically developing full term infants and from that of our children with MM. Because temperament is believed to be influenced by heredity, biology, and experience, parenting must also be considered as a variable (moderator) in temperament development. This concept of "goodness-of-fit" plays an important role in the parents' development, their perception of the infant, and the ultimate quality of the parent-child interactions [72].

Learning, for the child with MM, certainly takes place beyond the confines of preschool and classroom. Negotiating physicians, curious strangers, medical emergencies, educational diagnosticians, environmental barriers, and other stressors is a continual process. Raising a happy, self-reliant, and resilient child is the dream of parents. Perrin [73] has suggested that "adjustment" might be conceptualized as 1] the presence of fewer behavioral problems, 2] inter-personal functioning or competence, and 3] development of a positive self-concept. Parental perceptions of their child's actions and reactions contribute greatly to the moderation of, or exacerbation of, affective differences. It is incumbent on the professional to accurately discriminate between temperament characteristics that are barriers to inter-personal optimal functioning and truly psychiatric disorders. Given the neuro-anatomical and neuro-endocrine risks associated with MM, heightened awareness and assessment of temperament in this discriminating process is important.

## Conclusion

T.S. Eliot wrote,

We shall not cease from exploration

And the end of all our exploring

Will be to arrive where we started

*And know the place for the first time*.

As we continue, on behalf of children and youth with MM, to explore and better understand the cognitive and learning differences within the group, the developmental variations related to underlying central nervous system structures may continue to hamper the human tendency to categorize. The modal profile may not be singular.

As assessments of strengths and differences continue; as descriptors of quality of life are derived; as personality variances are accounted for; as curricula and "home programs" are constructed, the bio-psycho-social model increasingly becomes the clear route for professional and family partnership. For the researcher trying to join un-connected dots into a cohesive whole, we, like Eliot, may need to return to the early components long-standing in the evolution of the brain and its function. The ancient limbic and hypothalamic systems leave indelible tell-tale hints that should not be left behind in our quest for understanding and raising the resilient child.

## List of abbreviations

MM, myelomeningocele; HC, hydrocephalus

## Competing interests

The author(s) declare that they have no competing interests.

## Authors' contributions

Both authors contributed equally to this work. All authors read and approved the final manuscript.
